# Enantioselective Organocatalysis-Based Synthesis of 3-Hydroxy Fatty Acids and Fatty γ-Lactones

**DOI:** 10.3390/molecules24112081

**Published:** 2019-05-31

**Authors:** Asimina Bourboula, Dimitris Limnios, Maroula G. Kokotou, Olga G. Mountanea, George Kokotos

**Affiliations:** Department of Chemistry, National and Kapodistrian University of Athens, Panepistimiopolis, Athens 15771, Greece; minabour@chem.uoa.gr (A.B.); dlimnios@chem.uoa.gr (D.L.); mkokotou@chem.uoa.gr (M.G.K.); olgamount@chem.uoa.gr (O.G.M.)

**Keywords:** deuterated fatty acids, epoxides, hydroxy fatty acids, γ-lactones, organocatalysis

## Abstract

3-Hydroxy fatty acids have attracted the interest of researchers, since some of them may interact with free fatty acid receptors more effectively than their non-hydroxylated counterparts and their determination in plasma provides diagnostic information regarding mitochondrial deficiency. We present here the development of a convenient and general methodology for the asymmetric synthesis of 3-hydroxy fatty acids. The enantioselective organocatalytic synthesis of terminal epoxides, starting from long chain aldehydes, is the key-step of our methodology, followed by ring opening with vinylmagnesium bromide. Ozonolysis and subsequent oxidation leads to the target products. MacMillan’s third generation imidazolidinone organocatalyst has been employed for the epoxide formation, ensuring products in high enantiomeric purity. Furthermore, a route for the incorporation of deuterium on the carbon atom carrying the hydroxy group was developed allowing the synthesis of deuterated derivatives, which may be useful in biological studies and in mass spectrometry studies. In addition, the synthesis of fatty γ-lactones, corresponding to 4-hydroxy fatty acids, was also explored.

## 1. Introduction

Saturated and unsaturated fatty acids (FAs), as well as hydroxy fatty acids (HFAs) are components of biomolecules and have been shown to exert cellular effects through G protein–coupled receptors named free fatty acid receptors (FFA1–FFA4) and hydroxycarboxylic acid receptors (HCA1–HCA3) [1]. A recent study has shown that the G protein-coupled receptor 84 (GPR84) is activated by 2-hydroxy or 3-hydroxy medium chain fatty acids more effectively than by their non-hydroxylated counterparts [2]. 3-Hydroxy fatty acids are produced in mitochondria by β-oxidation [3] and are constituents of inflammatory lipopolysaccharides [4]. The determination of 3-hydroxy fatty acids in plasma or tissue cultures provides diagnostic information regarding medium/short-chain mitochondrial L-3 hydroxyacyl coenzyme A dehydrogenases (M/SCHAD) deficiency [5]. Recently, a method for the quantitative profiling of 3-hydroxy fatty acids as environmental markers of endotoxin in occupational and environmental samples has been developed, using liquid chromatography coupled to tandem mass spectrometry [6].

Several methods for the synthesis of 3-hydroxy acids are known in the literature. For example, these hydroxy acids may be synthesized by the reduction of β-keto esters, while the choice of the reducing agent may lead either to racemic or to chiral products [7,8]. An efficient synthesis of 3-hydroxy carboxylic acids employs an aldol-type reaction of unmasked iodoacetic acid with carbonyl compounds promoted by samarium iodide [9]. Another approach based on the Reformatsky reaction has been applied for the synthesis of medium chain β-hydroxy esters [10]. Recently, the synthesis of 3-hydroxylauric acid by γ-alkylation of doubly deprotonated ethyl acetoacetate followed by reduction of the β-keto moiety and ester hydrolysis has been reported [11]. 3-Hydroxy fatty acids may be also synthesized via the opening of chiral long chain terminal epoxides by Me_3_SiCN in the presence of TBAF, followed by hydrolysis of the resulting nitriles [12]. Another approach employs the ring opening of chiral 2-(2-benzyloxyethyl)-oxirane by a long chain alkylmagnesium bromide, followed by functional group transformations to give 3-hydroxy fatty acids [13]. The aim of the present study was the development of a convenient general methodology for the asymmetric synthesis of 3-hydroxy fatty acids. An organocatalytic step using an imidazolidinone catalyst was employed for the induction of chirality. In addition, a synthetic route to deuterated 3-hydroxy fatty acids is presented. The synthesis of 4-hydroxy fatty acids was also studied and applied to the synthesis of a fatty acid ester of a 4-hydroxy fatty acid.

## 2. Results and Discussion

Organocatalysis has been recognized as a powerful methodology in asymmetric catalysis, complementing metal catalysis and biocatalysis and offering new opportunities for the enantioselective synthesis of biomolecules [14]. The organocatalytic formation of epoxides was selected as the key-step of our asymmetric method, employing MacMillan’s imidazolidinone catalyst, which has found a wide range of applications [15]. A number of different enantioselective methodologies for the α-chlorination of aldehydes have been reported in literature, and the enantiomeric excess of the product depends on the catalyst, the chlorination agent and the reaction conditions [16,17,18,19,20]. For the chlorination of long chain aldehydes, we selected a modified procedure employing (2*R*,5*S*)-2-(*tert*-butyl)-3,5-dimethylimidazolidin-4-one trifluoroacetate as the organo-catalyst and 2,3,4,5,6,6-hexachlorocyclohexa-2,4-dien-1-one as the chlorination agent [21].

**Figure 1:**
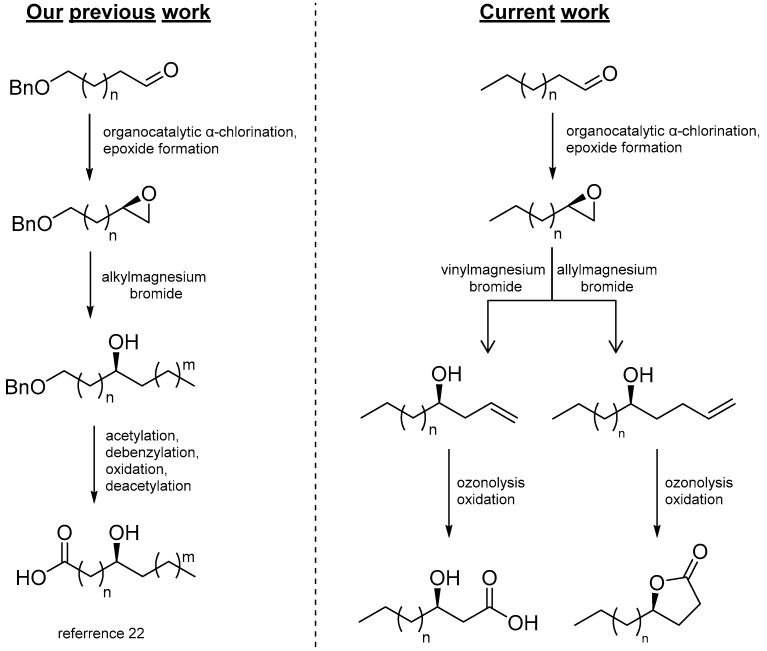
Previous work for the synthesis of 9-hydroxy fatty acids [22] (**left**) and current design plan for the synthesis of 3-hydroxy fatty acids or 4-hydroxy fatty acid derivatives (**right**).

We have recently presented the synthesis of 9-hydroxy fatty acids starting from monoprotected diols and employing organocatalytic α-chlorination, epoxide formation and ring opening by an alkylmagnesium bromide (Figure 1, left). Through a multistep procedure (acetylation, debenzylation, oxidation, deacetylation), 9-hydroxy fatty acids were obtained [22]. In the current work, we started from commercially available long chain aldehydes and through an organocatalytic α-chlorination, chiral terminal epoxides were synthesized, which were then opened either by vinylmagnesium bromide or by allylmagnesium bromide (Figure 1, right). A different strategy, employing ozonolysis and subsequent oxidation of the hydroxy alkenes, was followed to obtain the final 3-hydroxy fatty acids or 4-hydroxy fatty acid derivatives.

**Scheme 1:**
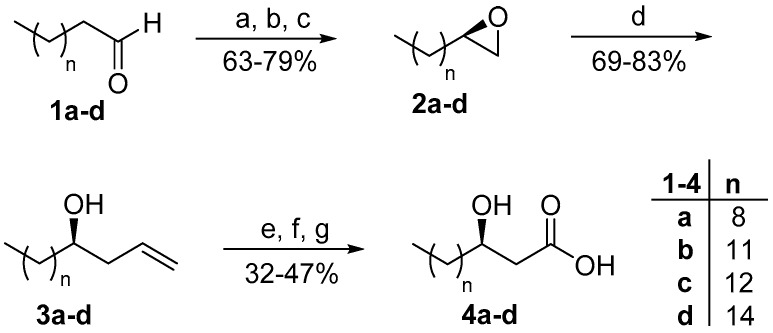
*Reagents and conditions:* (a) 2,3,4,5,6,6-hexachlorocyclohexa-2,4-dien-1-one, (2*R*,5*S*)-2-(*tert*-butyl)-3,5-dimethylimidazolidin-4-one trifluoroacetate (20%), THF, r.t., 15 min, (b) NaBH_4_, EtOH, 0 °C, 30 min, (c) KOH/EtOH/H_2_O, r.t., 15 min, (d) vinylmagnesium bromide 1M, CuI, dry THF, −78 °C, overnight, (e) O_3_, CH_2_Cl_2_/pyridine 9:1, −78 °C, (f) i. Ar, 5 min, ii. Me_2_S, 0 °C, 1 h, (g) i. MeCN, *t*-BuOH, H_2_O, 2-methylbut-2-ene, NaH_2_PO_4_, NaClO_2_/H_2_O, r.t., 2 h, ii. Na_2_SO_3_.

As depicted in Scheme 1, applying the selected protocol, commercially available long chain aldehydes **1a**–**d** were chlorinated and subsequent reduction by NaBH_4_, followed by treatment with KOH, led in a one-pot procedure to the formation of terminal chiral epoxides **2a**–**d** in good yields and high enantiomeric purity. Treatment of these epoxides with vinylmagnesium bromide resulted in the ring opening and afforded 4-hydroxy terminal alkenes **3a**–**d** in high yields**.** The target compounds **4a**–**d**, were obtained through ozonolysis of the alkene, followed by a Pinnick oxidation (Scheme 1).

3-Hydroxy fatty acids are of high biological importance and thus, their deuterated analogs could be useful in biology and in mass spectrometry as internal standards. When a hydrogen atom of a bioactive compound is replaced by deuterium, the deuterated derivative may present altered metabolic stability [23].

**Scheme 2:**
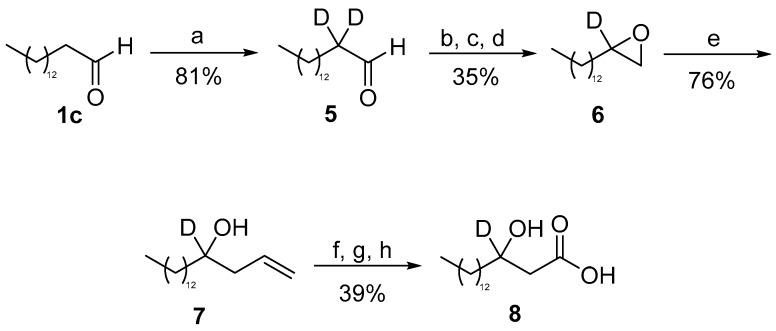
*Reagents and conditions:* (a) D_2_O, Et_3_N, 100 °C, 1 h, (b) *N*-chlorosuccinimide, d,l-proline, CH_2_Cl_2_, r.t., overnight, (c) NaBH_4_, EtOH, 0 °C, 15 min, (d) KOH/EtOH/H_2_O, r.t., 30 min, (e) vinylmagnesium bromide 1M, CuI, dry THF, −78 °C, overnight, (f) O_3_, CH_2_Cl_2_/pyridine 9:1, −78 °C, (g) i. Ar, 5 min, ii. Me_2_S, 0 °C, 1 h, (h) i. MeCN, *t*-BuOH, H_2_O, 2-methylbut-2-ene, NaH_2_PO_4_, NaClO_2_/H_2_O, r.t., 2 h, ii. Na_2_SO_3_.

Recently the FDA has approved the first deuterated drug [24], signifying a new era for research on deuterated bioactive compounds. Thus, we developed a method for the incorporation of a deuterium at the 3-position on the carbon atom carrying the hydroxy group. Our approach for the synthesis of deuterated 3-hydroxy palmitic acid is shown in Scheme 2. Deuterated epoxide **6** was obtained in low yield. Apparently the replacement of the deuterium by chlorine occurs in lower yield in comparison to the replacement of hydrogen.

**Figure 2:**
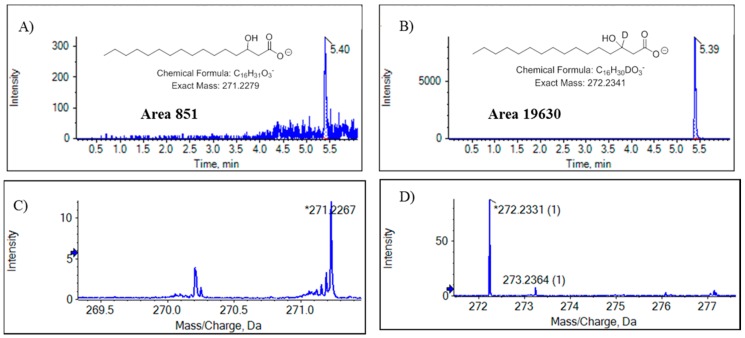
Extracted ion chromatograms for *m*/*z* 272.2341 (**A**) and *m*/*z* 271.2279 (**B**) and their corresponding mass spectra (**C**) and (**D**).

First, pentadecanal **1c** was converted into the corresponding α,α-dideuterated derivative **5** by treatment with D_2_O in the presence of triethylamine [25]. Organocatalytic α-chlorination of **5**, followed by reduction with NaBH_4_ and subsequent treatment with KOH, afforded in a one-pot procedure the deuterated epoxide **6**. For the α-chlorination, d,l-proline used as the catalyst and *N*-chlorosuccinimide as the chlorinating agent, since deuterated derivatives used as internal standards in mass spectrometry studies are not needed to be chiral compounds. Then, using similar reactions with those previously described, the deuterated hydroxy alkene **7** was obtained and converted into product **8** (Scheme 2). The degree of deuteration in compound **8** was determined using liquid chromatography-high resolution mass spectrometry (LC/HRMS) as depicted in Figure 2. The extracted ion chromatograms for *m*/*z* 272.2341 (corresponding to deuterated derivative **8**) and *m*/*z* 271.2279 (corresponding to non-deuterated analog of **8**) are shown in Figure 2A,B, while Figure 2C,D present the corresponding mass spectra. The deuteration degree of compound **8** was estimated to be 95.8%.

**Scheme 3:**
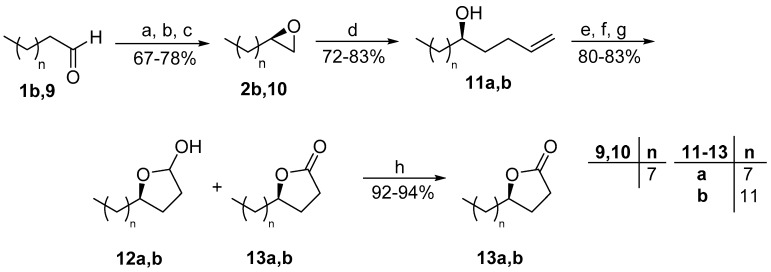
*Reagents and conditions:* (a) 2,3,4,5,6,6-hexachlorocyclohexa-2,4-dien-1-one, (2*R*,5*S*)-2-(*tert*-butyl)-3,5-dimethylimidazolidin-4-one trifluoroacetate (20%), THF, r.t., 15 min, (b) NaBH_4_, EtOH, 0 °C, 30 min, (c) KOH/EtOH/H_2_O, r.t., 15 min, (d) allylmagnesium bromide 1M, CuI, dry THF, −40 °C, 1 h, (e) O_3_, CH_2_Cl_2_, −78 °C, (f) i. O_2_, 5 min., ii. Ar, 5 min, (g) PPh_3_, dry CH_2_Cl_2_, r.t., overnight, (h) Jones oxidation.

To extend our studies and in an effort to synthesize 4-hydroxy fatty acids derivatives, a similar procedure was explored. As presented in Scheme 3, the ring opening of epoxides **2b** and **10** was carried out by treatment with allylmagnesium bromide to afford 5-hydroxy terminal alkenes **11a**,**b** in high yields. Ozonolysis of these compounds, followed by treatment with PPh_3_ led to a mixture of lactols **12a**,**b** and lactones **13a**,**b**. Jones oxidation of such mixtures resulted in full conversion to lactones **13a**,**b**. Both lactones are naturally occurring products found either in insects or in food stuff [26,27,28].

**Scheme 4:**
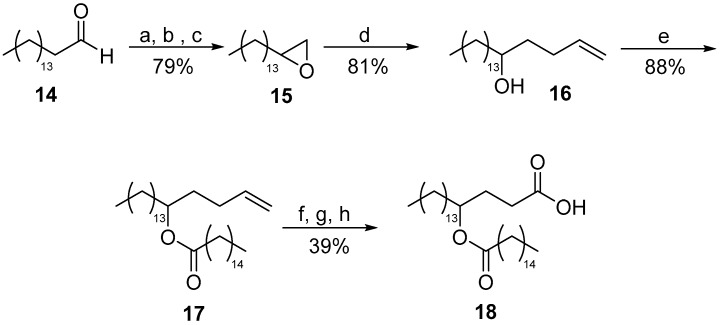
*Reagents and conditions:* (a) *N*-chlorosuccinimide, d,l-proline, CH_2_Cl_2_, r.t., overnight, (b) NaBH_4_, EtOH, 0 °C, 15 min, (c) KOH/EtOH/H_2_O, r.t., 30 min, (d) allylmagnesium bromide 1M, CuI, dry THF, −40 °C, 1 h, (e) CH_3_(CH_2_)_14_COOH, EDC, Et_3_N, 4-DMAP, dry CH_2_Cl_2_, r.t., overnight, (f) O_3_, CH_2_Cl_2_/pyridine 9:1, −78 °C, (g) i. Ar, ii. Me_2_S, 0 °C, 1 h, (h) i. MeCN, *t*-BuOH, H_2_O, 2-methylbut-2-ene, NaH_2_PO_4_, NaClO_2_/H_2_O, r.t., 2 h, ii. Na_2_SO_3_.

To avoid lactonization, the hydroxyl functionality of 5-hydroxy terminal alkenes has to be derivatized. Such a synthesis leading to biologically relevant 4-(palmitoyloxy)octadecanoic acid (4-PAHSA) is shown in Scheme 4. Fatty acid esters of hydroxy fatty acids (FAHFAs) have been recently identified as a novel class of endogenous lipids that present antidiabetic and/or anti-inflammatory properties [29,30,31]. Various families of FAHFAs varying in the chain length and the position of the ester group (5-, 9-, etc.) have been reported and synthetic routes to FAHFAs and HFAs, where the hydroxyl is at a position higher to 5-, have been developed [22,32,33]. FAHFAs containing the ester group at the 4-position have not yet reported and thus, we decided to synthesize such a derivative. Epoxide **15** was synthesized using d,l-proline as the catalyst and *N*-chlorosuccinimide for the α-chlorination step, followed by reduction with NaBH_4_ and treatment with KOH. Treatment of **15** with allylmagnesium bromide afforded hydroxy alkene **16** in high yield, which was coupled with palmitic acid, using *N*-ethyl-*N*′-(3-dimethylaminopropyl)carbodiimide (EDC) as the coupling agent in the presence of 4-(dimethylamino)pyridine (DMAP). Ozonolysis of **17**, followed by a Pinnick oxidation, as previously described, gave 4-PAHSA **18**.

## 3. Experimental Section

### 3.1. General Information

All reactions were monitored by analytical thin-layer chromatography (TLC) using plates precoated with silica gel (0.2 mm, 60 Å pore size) impregnated with a fluorescent indicator (254 nm). TLC plates were visualized by exposure to ultraviolet light (UV) and then stained with 12-molybdophosphoric acid hydrate in EtOH followed by brief heating on a hot plate.

^1^H-NMR spectra were recorded in CDCl_3_ at 200 MHz (Mercury, Varian, Darmstadt, Germany). Data were reported as follows: chemical shifts in ppm from TMS with the solvent resonance as the internal standard (CDCl_3_, *δ* 7.26 ppm), multiplicity (s = singlet, d = doublet, t = triplet, q = quartet, m = multiplet, br = broad signal), coupling constants (Hz) and integration. Proton-decoupled carbon nuclear magnetic resonance spectra (^13^C-NMR) were recorded at 50 MHz. Chemical shifts were expressed in parts per million (ppm, *δ* scale) downfield from TMS and are referenced to the carbon resonances of the solvent (CDCl_3_, *δ* 77.0). High-resolution mass spectra (HRMS) were recorded on a Maxis Impact QTOF (Bruker, Bremen, Germany) and a 4600 Triple TOF (AB Sciex, Singapore). Optical rotations ([α]_D_^20^) were measured on an AA-65 series (Optical Activity Ltd., Bury, UK) polarimeter and melting points (m.p.) were measured on a Buchi 530 apparatus (Buchi, Flawil, Switzerland).

Enantiomeric excesses of **4a**–**d** were determined by chiral LC/HRMS analysis using a Chiralpak AD-RH column and acetonitrile/H_2_O (95:5) containing 0.1% formic acid as the mobile phase (flow rate 0.3 mL/min). Extracted ion chromatograms for the determination of enantiomeric excesses of **4a**–**d** are presented in the Supplementary Materials.

### 3.2. Synthesis and Characterization

#### 3.2.1. General Procedure for the Synthesis of Chiral Epoxides **2a**–**d**, **10**

To a solution of (2*R*,5*S*)-2-(*tert*-butyl)-3,5-dimethylimidazolidin-4-one trifluoroacetate (57 mg, 0.20 mmol) in THF (0.5 mL), 2,3,4,5,6,6-hexachlorocyclohexa-2,4-dien-1-one (331 mg, 1.10 mmol) was added and the solution was stirred for 5 min, before the aldehyde (1.00 mmol) was added. The reaction mixture was stirred for 30 min at room temperature and was cooled to 0 °C, before EtOH (0.6 mL) and NaBH_4_ (121 mg, 3.20 mmol) were added. After 10 min, the medium was warmed to room temperature for 5 min, before a freshly prepared solution of aqueous KOH (1.70 g KOH diluted in 2.7 mL water) and EtOH (1.3 mL) were added and the resulting mixture was stirred vigorously for 30 min. Then, water (1 × 20 mL) was added to the mixture before it was extracted with Et_2_O (3 × 10 mL), washed with brine (1 × 20 mL), dried over Na_2_SO_4_, filtered and concentrated under reduced pressure. Purification of the resulting residue by silica gel chromatography (petroleum ether 40–60 °C/diethyl ether, 9:1 or 8:2) afforded the title compounds.

*(R)-2-Nonyloxirane* (**2a**). Colorless oil, 129 mg, yield: 76%; R_f_ value: (pet. ether:diethyl ether = 9:1) 0.42; [α]_D_^20^ −5.80 (*c* 1.0, CHCl_3_), [ref. [18], for *S*-enantiomer: [α]_D_^20^ +6.34 (*c* 1.0, CHCl_3_)]; ^1^H-NMR (CDCl_3_) *δ* 2.85–2.76 (m, 1H, OCH), 2.64 (dd, *J* = 5.0, 4.1 Hz, 1H, OC*H*H), 2.36 (dd, *J* = 5.1, 2.7 Hz, 1H, OC*H*H), 1.64–0.98 (m, 16H, 8 × CH_2_), 0.81 (t, *J* = 6.3 Hz, 3H, CH_3_); ^13^C-NMR (CDCl_3_) *δ* 52.0, 46.7, 32.4, 31.7, 29.4, 29.3, 29.2, 25.8, 22.5, 13.9.

*(R)-2-Dodecyloxirane* (**2b**). Colorless oil, 174 mg, yield 82%; R_f_ value: (pet. ether:diethyl ether = 9:1) 0.37; [α]_D_^20^ +4.70 (*c* 1.0, CHCl_3_), [ref. [34], [α]_D_^20^ +4.30 (c 1.42, CHCl_3_)]; ^1^H-NMR (CDCl_3_) *δ* 2.92–2.84 (m, 1H, OCH), 2.72 (dd, *J* = 5.0, 4.0 Hz, 1H, OC*H*H), 2.44 (dd, *J* = 5.1, 2.7 Hz, 1H, OC*H*H), 1.57–1.16 (m, 22H, 11 × CH_2_), 0.86 (t, *J* = 6.2 Hz, 3H, CH_3_); ^13^C-NMR (CDCl_3_) *δ* 52.5, 47.2, 32.7, 32.1, 29.8, 29.7, 29.6, 26.2, 22.9, 14.3.

*(R)-2-Tridecyloxirane* (**2c**). Colorless oil, 179 mg, yield 79%; R_f_ value: (pet. ether:diethyl ether = 9:1) 0.31; [α]_D_^20^ +5.70 (*c* 0.5 CHCl_3_), [ref. [35], [α]_D_^20^ +6.54 (*c* 1.0, CHCl_3_)]; ^1^H-NMR (CDCl_3_) *δ* 2.79–2.70 (m, 1H, OCH), 2.57 (dd, *J* = 5.9, 4.0 Hz, 1H, OC*H*H), 2.29 (dd, *J* = 5.1, 2.7 Hz, 1H, OC*H*H), 1.48–1.05 (m, 24H, 12 × CH_2_), 0.77 (t, *J* = 5.4 Hz, 3H, CH_3_); ^13^C-NMR (CDCl_3_) *δ* 51.8, 46.5, 32.4, 31.7, 29.5, 29.4, 29.3, 29.2, 25.8, 22.5, 13.8.

*(R)-2-Pentadecyloxirane* (**2d**). Colorless oil, 211 mg, yield 83%; R_f_ value: (pet. ether:diethyl ether = 8:2) 0.37; [α]_D_^20^ +5.00 (*c* 1.0, CHCl_3_), [ref. [36], [α]_D_^20^ +5.43 (*c* 1.4, CHCl_3_)]; ^1^H-NMR (CDCl_3_) *δ* 2.89–2.80 (m, 1H, OCH), 2.67 (dd, *J* = 5.0, 4.0 Hz, 1H, OC*H*H), 2.39 (dd, *J* = 5.1, 2.7 Hz, 1H, OC*H*H), 1.53–1.11 (m, 28H, 14 × CH_2_), 0.83 (t, *J* = 5.4 Hz, 3H, CH_3_); ^13^C-NMR (CDCl_3_) *δ* 52.1, 46.8, 32.4, 31.9, 29.6, 29.5, 29.4, 29.3, 25.9, 22.6, 14.0.

*(R)-2-Octyloxirane* (**10**). Colorless oil, 117 mg, yield 75%; R_f_ value: (pet. ether:diethyl ether = 9:1) 0.35; [α]_D_^20^ +8.00 (*c* 1.0, CHCl_3_) [ref. [37], for *S*-enantiomer: [α]_D_^20^ −8.20 (*c* 1.03, CHCl_3_)]; ^1^H-NMR (CDCl_3_) *δ* 2.89–2.80 (m, 1H, OCH), 2.68 (dd, *J* = 5.0, 4.0 Hz, 1H, OC*H*H), 2.40 (dd, *J* = 5.1, 2.7 Hz, 1H, OC*H*H), 1.53–1.11 (m, 14H, 7 × CH_2_), 0.83 (t, *J* = 6.4 Hz, 3H, CH_3_); ^13^C-NMR (CDCl_3_) *δ* 52.2, 46.9, 32.4, 31.7, 29.4, 29.3, 29.1, 25.9, 22.5, 13.9.

#### 3.2.2. General Procedure for the Synthesis of Racemic Epoxides **6**, **15**

To a solution of **5** or **14** (1.00 mmol) in CH_2_Cl_2_ (5 mL), d,l-proline (115 mg, 0.10 mmol) and *N*-chlorosuccinimide (174 mg, 1.30 mmol) were added and the reaction mixture was stirred for 16 h at room temperature. The reaction mixture was cooled to 0 °C, before NaBH_4_ (121 mg, 3.20 mmol) and EtOH (1 mL) were added. After 10 min, the medium was warmed to room temperature for 5 min before a freshly prepared solution of aqueous KOH (2.25 g KOH diluted in 3.4 mL distilled water) and EtOH (1.7 mL) were added and the resulting mixture was stirred vigorously for 30 min. At this stage, water (10 mL) was added to the mixture before it was extracted with Et_2_O (1 × 10 mL), saturated aqueous solution of NH_4_Cl (1 × 10 mL) and brine (1 × 10 mL). The combined organic layers were dried over Na_2_SO_4_ and concentrated in vacuo. The resulting residue purified by column chromatography (petroleum ether 40–60 °C/diethyl ether, 9:1 or 8:2).

*2-Tridecyloxirane-2-d* (**6**). Colorless oil, 80 mg, yield 35%; R_f_ value: (pet. ether:diethyl ether = 8:2) 0.48; ^1^H-NMR (CDCl_3_) *δ* 2.68 (d, *J* = 5.0 Hz, 1H, OC*H*H), 2.40 (d, *J* = 5.0 Hz, 1H, OC*H*H), 1.53–1.03 (m, 24H, 12 × CH_2_), 0.84 (t, *J* = 6.2 Hz, 3H, CH_3_); ^13^C-NMR (CDCl_3_) *δ* 51.8 (t, *J* = 25.0 Hz, OCD), 46.8, 32.3, 31.9, 29.6, 29.5, 29.4, 29.3, 25.9, 22.6, 14.0; HRMS (ESI) exact mass calculated for [M + Na]^+^ (C_15_H_29_DONa^+^) requires *m*/*z* 250.2252, found *m*/*z* 250.2249.

*2-Tetradecyloxirane* (**15**). Colorless oil, 195 mg, yield 81%; R_f_ value: (pet. ether:diethyl ether = 9:1) 0.41; ^1^H-NMR (CDCl_3_) *δ* 2.87–2.79 (m, 1H, OCH), 2.66 (dd, *J* = 5.1, 4.0 Hz, 1H, OC*H*H), 2.38 (dd, *J* = 6.0, 2.0 Hz, 1H, OC*H*H), 1.58–0.98 (m, 26H, 13 × CH_2_), 0.83 (t, *J* = 6.4 Hz, 3H, CH_3_); ^13^C-NMR (CDCl_3_) *δ* 52.1, 46.8, 32.4, 31.8, 29.6, 29.5, 29.4, 29.3, 25.9, 22.6, 13.9 [38].

#### 3.2.3. General Procedure for the Synthesis of 4-Hydroxy Terminal Alkenes **3a**–**d**, **7**

To a flame-dried round flask, degassed and under argon atmosphere, copper(I) iodide (38 mg, 0.20 mmol) and vinylmagnesium bromide (1M solution in THF, 2.0 mL, 2.00 mmol) were added and cooled to −78 °C. Then, a solution of the epoxide (1.00 mmol) in anhydrous THF (5 mL) was added dropwise and the reaction mixture was left stirring at −78 °C overnight. The reaction mixture was allowed to warm to room temperature and after treatment with a saturated aqueous solution of NH_4_Cl (1 × 5 mL) was extracted with Et_2_O (3 × 10 mL). The combined organic layers were washed with brine (1 × 30 mL), dried over Na_2_SO_4_, filtered and concentrated under reduced pressure. Purification of the resulting residue by silica gel chromatography (petroleum ether 40–60 °C/diethyl ether, 9:1 or 8:2) afforded the desired compounds.

*(R)-Tridec-1-en-4-ol* (**3a**). Colorless oil, 169 mg, yield 85%; R_f_ value: (pet. ether:diethyl ether = 8:2) 0.48; [α]_D_^20^ −6.70 (*c* 1.0, CHCl_3_) [ref. [39], [α]_D_^20^ −7.50 (*c* 1.0, CHCl_3_)]; ^1^H-NMR (CDCl_3_) *δ* 5.87–5.68 (m, 1H, =CH), 5.15–5.04 (m, 2H, =CH_2_), 3.66–3.54 (m, 1H, C*H*OH), 2.32–2.02 (m, 3H, C*H_2_*CH, OH), 1.49–1.09 (m, 16H, 8 × CH_2_), 0.84 (t, *J* = 6.3 Hz, 3H, CH_3_); ^13^C-NMR (CDCl_3_) *δ* 134.9, 117.7, 70.6, 41.8, 36.7, 31.8, 29.6, 29.5, 29.3, 25.6, 22.6, 14.0; HRMS (ESI) exact mass calculated for [M + Na]^+^ (C_13_H_26_ONa^+^) requires *m*/*z* 221.1876, found *m*/*z* 221.1875.

*Hexadec-1-en-4-ol* (**3b**). Colorless oil, 195 mg, yield 81%; R_f_ value (pet. ether/diethyl ether 9:1) 0.52; [α]_D_^20^ −5.00 (*c* 1.0, CHCl_3_) [ref. [40], [α]_D_^20^ −5.50 (*c* 1.0, CHCl_3_)]; ^1^H-NMR (CDCl_3_) *δ* 5.92–5.71 (m, 1H, =CH), 5.17–5.06 (m, 2H, =CH_2_), 3.67–3.51 (m, 1H, C*H*OH), 2.34–2.01 (m, 2H, C*H_2_*CH), 1.83 (s, 1H, OH), 1.48–0.96 (m, 22H, 11 × CH_2_), 0.86 (t, *J* = 6.3 Hz, 3H, CH_3_); ^13^C-NMR (CDCl_3_) *δ* 134.9, 117.8, 70.6, 41.9, 36.8, 31.9, 29.6, 29.3, 25.6, 22.6, 14.0; HRMS (ESI) exact mass calculated for [M + Na]^+^ (C_16_H_32_ONa^+^) requires *m*/*z* 263.2345, found *m*/*z* 263.2340 [41].

*(R)-Heptadec-1-en-4-ol* (**3c**). Colorless oil, 211 mg, yield 83%; R_f_ value (pet. ether:diethyl ether = 9:1) 0.46; [α]_D_^20^ + 4.00 (*c* 1.0, CHCl_3_), [ref. [35], [α]_D_^20^ +4.92 (*c* 1.0, CHCl_3_)]; ^1^H-NMR (CDCl_3_) *δ* 5.90–5.70 (m, 1H, =CH), 5.12–5.05 (m, 2H, =CH_2_), 3.68–3.53 (m, 1H, C*H*OH), 2.32–2.02 (m, 3H, C*H_2_*CH, OH), 1.49–1.10 (m, 24H, 12 × CH_2_), 0.85 (t, *J* = 6.3 Hz, 3H, CH_3_); ^13^C-NMR (CDCl_3_) *δ* 134.9, 117.7, 70.6, 41.9, 36.7, 31.9, 29.6, 29.3, 25.6, 22.6, 14.0; HRMS (ESI) exact mass calculated for [M + Na]^+^ (C_17_H_34_ONa^+^) requires *m*/*z* 277.2502, found *m*/*z* 277.2503.

*(R)-Nonadec-1-en-4-ol* (**3d**). White solid, 220 mg, m.p.: 50–52 °C (ref. [42], 44–45 °C), yield 79%; R_f_ value (pet. ether:diethyl ether = 8:2) 0.57; [α]_D_^20^ −17.0 (*c* 0.50, CHCl_3_), [ref. [43], [α]_D_^20^ −18.33 (*c* 0.36, CHCl_3_)]; ^1^H-NMR (CDCl_3_) *δ* 5.90–5.69 (m, 1H, =CH), 5.12–5.04 (m, 2H, =CH_2_), 3.64–3.53 (m, 1H, C*H*OH), 2.32–2.03 (m, 3H, C*H_2_*CH, OH), 1.41–1.09 (m, 28H, 14 × CH_2_), 0.85 (t, *J* = 5.8 Hz, 3H, CH_3_); ^13^C-NMR (CDCl_3_) *δ* 138.6, 114.6, 71.3, 37.4, 36.4, 31.9, 30.0, 29.7, 29.3, 25.6, 22.6, 14.0; HRMS (ESI) exact mass calculated for [M + Na]^+^ (C_19_H_38_ONa^+^) requires *m*/*z* 305.2815, found *m*/*z* 305.2811.

*Heptadec-1-en-4-d-4-ol* (**7**). Colorless oil, 194 mg, yield 76%; R_f_ value (pet. ether:diethyl ether = 7:3) 0.52; ^1^H-NMR (CDCl_3_) *δ* 5.91–5.70 (m, 1H, =CH), 5.13–5.05 (m, 2H, =CH_2_), 2.31–1.99 (m, 3H, C*H_2_*CH, OH), 1.41–1.14 (m, 24H, 12 × CH_2_), 0.85 (t, *J* = 6.3 Hz, 3H, CH_3_); ^13^C-NMR (CDCl_3_) *δ* 134.9, 117.8, 70.1 (t, *J* = 20 Hz), 41.7, 36.6, 31.9, 29.6, 29.3, 25.6, 22.6, 14.0; HRMS (ESI) exact mass calculated for [M + Na]^+^ (C_17_H_33_DONa^+^) requires *m*/*z* 278.2565, found *m*/*z* 278.2566.

#### 3.2.4. General Procedure for the Synthesis of 5-Hydroxy Terminal Alkenes **11a**,**b**, **16**

To a flame-dried round flask, degassed and under argon atmosphere, copper(I) iodide (38 mg, 0.20 mmol) and allylmagnesium bromide (1M solution in THF, 2.0 mL, 2.00 mmol) were added and cooled to −40 °C. Then, a solution of the epoxide (1.00 mmol) in anhydrous THF (5 mL) was added dropwise and the reaction mixture was left stirring at −40 °C for 2 h. The reaction mixture was allowed to warm to room temperature and after treatment with a saturated aqueous solution of NH_4_Cl (5 mL) was extracted with Et_2_O (3 × 10 mL). The combined organic layers were washed with brine (30 mL), dried over Na_2_SO_4_, filtered and concentrated under reduced pressure. Purification of the resulting residue by silica gel chromatography (petroleum ether 40–60 °C/diethyl ether, 9:1 or 8:2) afforded the desired products.

*(R)-Tridec-1-en-5-ol* (**11a**). Colorless oil, 151 mg, yield 76%; R_f_ value (pet. ether:diethyl ether = 9:1) 0.59; [α]_D_^20^ +2.30 (*c* 0.9, MeOH), [ref. [37], [α]_D_^20^ +2.40 (*c* 0.86, MeOH)]; ^1^H-NMR (CDCl_3_) *δ* 5.92–5.72 (m, 1H, =CH), 5.06–4.92 (m, 2H, =CH_2_), 3.63–3.55 (m, 1H, C*H*OH), 2.25–2.04 (m, 2H, C*H_2_*CH=), 1.94 (s, 1H, OH), 1.55–1.15 (m, 16H, 8 × CH_2_), 0.86 (t, *J* = 6.4 Hz, 3H, CH_3_); ^13^C-NMR (CDCl_3_) *δ* 138.6, 114.6, 71.4, 37.4, 36.4, 30.0, 29.7, 29.6, 29.2, 25.6, 25.5, 22.6, 22.4, 14.1.

*(R)-Heptadec-1-en-5-ol* (**11b**). Colorless oil, 193 mg, yield 76%; R_f_ value (pet. ether:diethyl ether = 9:1) 0.61; [α]_D_^20^ + 0.80 (*c* 0.5, CHCl_3_); ^1^H-NMR (CDCl_3_) *δ* 5.94–5.74 (m, 1H, =CH), 5.09–4.93 (m, 2H, =CH_2_), 3.67–3.55 (m, 1H, C*H*OH), 2.31–2.00 (m, 2H, C*H_2_*CH=), 1.58–1.15 (m, 25H, 12 × CH_2_, OH), 0.87 (t, *J* = 6.0 Hz, 3H, CH_3_); ^13^C-NMR (CDCl_3_) *δ* 138.6, 114.6, 71.4, 37.5, 36.4, 31.9, 31.7, 30.1, 29.6, 29.3, 25.6, 22.7, 14.1 [44].

*Nonadec-1-en-5-ol* (**16**). Colorless oil, 223 mg, yield 79%; R_f_ value (pet. ether:diethyl ether = 9:1) 0.53; ^1^H-NMR (CDCl_3_) *δ* 5.92–5.72 (m, 1H, =CH), 5.07–4.91 (m, 2H, =CH_2_), 3.64–3.53 (m, 1H, C*H*OH), 2.26–2.04 (m, 2H, C*H_2_*CH=), 1.88 (s, 1H, OH), 1.57–1.24 (m, 28H, 14 × CH_2_), 0.86 (t, *J* = 6.3 Hz, 3H, CH_3_); ^13^C-NMR (CDCl_3_) *δ* 138.6, 114.6, 71.3, 37.4, 36.4, 33.2, 33.1, 31.9, 30.0, 29.7, 29.5, 29.3, 25.6, 22.6, 14.0 [45].

#### 3.2.5. General Procedure for the Synthesis of 3-Hydroxy Fatty Acids **4a**–**d**, **8**

Ozone was passed through a solution of **3a**–**d** or **7** (1.00 mmol) in a mixture of CH_2_Cl_2_ (4.5 mL) and pyridine (0.5 mL) at −78 °C. Once the reaction solution had a persistent blue color, was Ar passed through until the solution was discolored, then dimethylsulfide (0.4 mL, 14.0 mmol) was added at 0 °C and the reaction mixture was left stirring for 1 to 2 h. After concentration under reduced pressure, the residue was oxidized to the desired 3-hydroxy fatty acid as described below.

To a round-bottomed flask containing the aldehyde (1.00 mmol), MeCN (10 mL), *t*-BuOH (2 mL), distilled H_2_O (0.5 mL), 2-methylbut-2-ene (0.5 mL), NaH_2_PO_4_ (48 mg, 0.40 mmol), aqueous solution of NaClO_2_ (154 mg NaClO_2_ diluted in 1 mL water) were added at 0 °C. The reaction mixture was left stirring for 2 h and then was quenched by the addition of Na_2_SO_3_ (101 mg, 0.80 mmol). After acidification to pH 1 by the addition of a solution of 1N HCl, the reaction mixture was extracted with Et_2_O (3 × 10 mL). The combined organic layers were washed with brine (30 mL), dried over Na_2_SO_4_ and concentrated in vacuo. The resulting residue was purified by column chromatography (CH_2_Cl_2_/MeOH 9:1 containing 10% CH_3_COOH) and recrystallized with hexane.

*(R)-3-Hydroxydodecanoic acid* (**4a**). White solid, 76 mg, m.p.: 63–64 °C (ref. [46], 67–68 °C), yield 35%; R_f_ value (dichloromethane:methanol = 9:1) 0.37; [α]_D_^20^ −15.80 (*c* 1.0, CHCl_3_), [ref. [47], [α]_D_^20^ −16.90 (*c* 1.0, CHCl_3_)]; ^1^H-NMR (CDCl_3_) *δ* 7.47 (s, 1H, COOH), 4.00 (s, 1H, C*H*OH), 2.55–2.34 (m, 2H, C*H_2_*COOH), 1.43–1.24 (m, 16H, 8 × CH_2_), 0.85 (t, *J* = 5.7 Hz, 3H, CH_3_); ^13^C-NMR (CDCl_3_) *δ* 177.3, 68.2, 41.2, 36.4, 31.8, 29.6, 29.5, 29.3, 25.4, 22.6, 14.0; HRMS (ESI) exact mass calculated for [M − H]^−^ (C_12_H_23_O_3_^−^) requires *m*/*z* 215.1653, found *m*/*z* 215.1634; LC-HRMS analysis: 95% ee, Chiralpak AD-RH, acetonitrile/H_2_O (95:5) formic acid 0.1%, 0.3 mL/min, t_R_ = 9.3 (minor), t_R_ = 9.9 (major).

*(R)-3-Hydroxypentadecanoic acid* (**4b**). White solid, 119 mg, m.p.: 82–83 °C (ref. [48], 86–87 °C), yield 46%; R_f_ value (dichloromethane:methanol = 9:1) 0.29; [α]_D_^20^ −14.00 (*c* 1.0, CHCl_3_), [ref. [49], [α]_D_^20^ −15.00 (*c* 1.0, CHCl_3_)]; ^1^H-NMR (CDCl_3_) *δ* 4.06–3.97 (m, 1H, C*H*OH), 2.63–2.46 (m, 2H, C*H_2_*COOH), 1.57–1.01 (m, 22H, 11 × CH_2_), 0.85 (t, *J* = 6.3 Hz, 3H, CH_3_); ^13^C-NMR (CDCl_3_) *δ* 177.4, 68.0, 41.0, 36.5, 31.9, 29.6, 29.6, 29.5, 29.4, 25.4, 22.7, 14.1; HRMS (ESI) exact mass calculated for [M − H]^−^ (C_15_H_29_O_3_^−^) requires *m*/*z* 257.2122, found *m*/*z* 257.2110; LC-HRMS analysis: 91% ee, Chiralpak AD-RH, acetonitrile/H_2_O (95:5) formic acid 0.1%, 0.3 mL/min, t_R_ = 12.7 (minor), t_R_ = 14.7 (major).

*(R)-3-Hydroxyhexadecanoic acid* (**4c**). White solid, 106 mg, m.p.: 80–81 °C (ref. [50], 83–84 °C), yield 39%; R_f_ value (dichloromethane:methanol = 9:1) 0.32; [α]_D_^20^ −13.00 (*c* 2.0, CHCl_3_), [ref. [13], [α]_D_^20^ −12.60 (*c* 2.08, CHCl_3_)]; ^1^H-NMR (CDCl_3_) *δ* 4.02 (s, 1H, C*H*OH), 2.61–2.31 (m, 3H, C*H_2_*COOH, OH), 1.66–1.11 (m, 24H, 12 × CH_2_), 0.87 (t, *J* = 6.3 Hz, 3H, CH_3_); ^13^C-NMR (CDCl_3_) *δ* 177.9, 68.0, 41.0, 36.4, 31.9, 29.7, 29.6, 29.5, 29.4, 29.2, 25.4, 22.7, 14.1; HRMS (ESI) exact mass calculated for [M − H]^−^ (C_16_H_31_O_3_^−^) requires *m*/*z* 271.2279, found *m*/*z* 271.2274; LC-HRMS analysis: 89% ee, Chiralpak AD-RH, acetonitrile/H_2_O (95:5) formic acid 0.1%, 0.3 mL/min, t_R_ = 14.7 (minor), t_R_ = 17.8 (major).

*(R)-3-Hydroxyoctadecanoic acid* (**4d**). White solid, 129 mg, m.p.: 82–84 °C (ref. [51], 88–90 °C), yield 43%; R_f_ value (dichloromethane:methanol = 9:1) 0.27; [α]_D_^20^ −16.00 (*c* 1.0, CHCl_3_), [ref. [8], [α]_D_^20^ −15.80 (*c* 1.0, CHCl_3_)]; ^1^H-NMR (CDCl_3_) *δ* 4.06–4.01 (m, 1H, C*H*OH), 2.62–2.39 (m, 3H, C*H_2_*COOH, OH), 1.65–1.17 (m, 28H, 14 × CH_2_), 0.87 (t, *J* = 6.4 Hz, 3H, CH_3_); ^13^C-NMR (CDCl_3_) *δ* 177.3, 68.0, 41.0, 36.5, 31.9, 29.7, 29.6, 29.5, 29.4, 25.4, 22.7, 14.1; HRMS (ESI) exact mass calculated for [M − H]^−^ (C_18_H_35_O_3_^−^) requires *m*/*z* 299.2592, found *m*/*z* 299.2572; LC-HRMS analysis: 92% ee, Chiralpak AD-RH, acetonitrile/H_2_O (95:5) formic acid 0.1%, 0.3 mL/min, t_R_ = 21.0 (minor), t_R_ = 26.5 (major).

*3-Hydroxyhexadecanoic-3-d acid* (**8**). White solid, 104 mg, m.p.: 80–82 °C, yield 38%; R_f_ value (pet. ether:diethyl ether = 7:3) 0.27; ^1^H-NMR (CDCl_3_) *δ* 2.58 (d, *J* = 16.0 Hz, 1H, C*H*HCOOH), 2.45 (d, *J* = 16.0 Hz, 1H, C*H*HCOOH), 2.03 (s, 1H, OH), 1.48–1.10 (m, 24H, 12 × CH_2_), 0.88 (t, *J* = 6.0 Hz, 3H, CH_3_); ^13^C-NMR (CDCl_3_) *δ* 177.3, 40.8, 36.4, 31.9, 29.7, 29.6, 29.5, 29.4, 25.4, 22.7, 14.1; HRMS (ESI) exact mass calculated for [M − H]^−^ (C_16_H_30_DO_3_^−^) requires *m*/*z* 272.2341, found *m*/*z* 272.2331.

#### 3.2.6. General Procedure for the Synthesis of Lactones **13a**,**b**

Ozone passed through a solution of **11a**,**b** (1.00 mmol) in CH_2_Cl_2_ (10 mL) at −78 °C. Once the reaction solution had a persisted blue color, O_2_ passed through for 5 min and then Ar, until the solution was discolored. The reaction mixture was allowed to warm to room temperature and then, PPh_3_ (262 mg, 1.00 mmol) was added and left stirring overnight. The solution was concentrated in vacuo and the resulting residue was purified by column chromatography (petroleum ether 40–60 °C/diethyl ether, 8:2 or 7:3). Thus, a mixture of lactol/lactone was obtained, which was fully oxidized to the desired lactone as described below.

To a solution of lactol/lactone (1.00 mmol) in acetone (10 mL) Jones solution 2 M (1.5 mL, 3.00 mmol) was added dropwise at 0 °C and the reaction mixture was left stirring for 1 h. Then, the reaction mixture was quenched by the addition of a saturated aqueous solution of NaHSO_3_ (10 mL) and extracted with Et_2_O (3 × 10 mL). The combined organic layers were washed with brine (30 mL), dried over Na_2_SO_4_ and concentrated in vacuo. The resulting residue was purified by column chromatography (petroleum ether 40–60 °C/diethyl ether, 7:3 or 6:4).

*(R)-5-Octyldihydrofuran-2(3H)-one* (**13a**). Colorless oil, 182 mg, yield 92%; R_f_ value (pet. ether:diethyl ether = 7:3) 0.62; [α]_D_^20^ + 42.80 (*c* 1.0, MeOH) [ref. [37], for *S*-enantiomer: [α]_D_^20^ −43.10 (*c* 1.01, MeOH)]; ^1^H-NMR (CDCl_3_) *δ* 4.52–4.39 (m, 1H, CH), 2.54–2.46 (m, 2H, CH_2_CO), 2.37–2.21 (m, 1H, C*H*HCH), 1.89–1.23 (m, 15H, 7 × CH_2_, C*H*HCH), 0.84 (t, *J* = 6.3 Hz, 3H, CH_3_); ^13^C-NMR (CDCl_3_) *δ* 177.4, 81.0, 35.5, 31.7, 29.3, 29.2, 29.1, 28.8, 27.9, 25.1, 22.6, 14.0.

*(R)-5-Dodecyldihydrofuran-2(3H)-one* (**13b**). White solid, 226 mg, m.p.: 35–36 °C (ref. [52], 38–39 °C), yield 89%; R_f_ value (pet. ether:diethyl ether = 7:3) 0.56; [α]_D_^20^ +7.60 (*c* 1.0, CHCl_3_); ^1^H-NMR (CDCl_3_) *δ* 4.56–4.39 (m, 1H, CH), 2.56–2.44 (m, 2H, CH_2_CO), 2.38–2.22 (m, 1H, C*H*HCH), 1.93–1.56 (m, 3H, CH_2_, C*H*HCH), 1.59–1.24 (m, 20H, 10 × CH_2_), 0.86 (t, *J* = 6.3 Hz, 3H, CH_3_); ^13^C-NMR (CDCl_3_) *δ* 177.3, 81.0, 35.5, 31.9, 29.6, 29.5, 29.4, 29.3, 28.8, 28.0, 25.2, 22.6, 14.1.

#### 3.2.7. Nonadec-1-en-5-yl Palmitate (**17**)

To a solution of palmitic acid (1.0 g, 4.00 mmol) in dry CH_2_Cl_2_ (15 mL), EDC (770 mg, 4.00 mmol), Et_3_N (0.6 mL, 4.00 mmol) and 4-DMAP (24 mg, 0.20 mmol) were added at 0 °C and the reaction mixture was left stirring for 10 min. Then, a solution of alcohol **16** (300 mg, 1.00 mmol) in dry CH_2_Cl_2_ (10 mL) was added and the mixture was left stirring at room temperature overnight. The reaction mixture was washed sequentially with a saturated aqueous solution of NH_4_Cl (10 mL), an aqueous solution of 10% NaHCO_3_ (10 mL), an aqueous solution of 10% citric acid (10 mL) and brine (15 mL). The combined organic layers were dried over Na_2_SO_4_, concentrated in vacuo and the product was purified by column chromatography (pet.ether 40–60 °C/ EtOAc 95:5). White solid; 458 mg, m.p.: 105–107 °C, yield 88%; R_f_ value (pet. ether:EtOAc = 95:5) 0.44; ^1^H-NMR (CDCl_3_) *δ* 5.88–5.70 (m, 1H, =CH), 5.05–4.83 (m, 3H, =CH_2_, CHCOO), 2.27 (t, *J* = 7.4 Hz, 2H, CH_2_COO), 2.10–2.00 (m, 2H, C*H_2_*CH=), 1.71–1.25 (m, 54H, 27 × CH_2_), 0.87 (t, *J* = 6.4 Hz, 6H, 2 × CH_3_); ^13^C-NMR (CDCl_3_) *δ* 173.5, 137.9, 114.8, 73.4, 34.7, 34.1, 33.4, 31.9, 29.7, 29.6, 29.5, 29.4, 29.3, 29.2, 25.3, 25.1, 22.7, 14.1; HRMS (ESI) exact mass calculated for [M + Na]^+^ (C_35_H_68_O_2_Na^+^) requires *m*/*z* 543.5112, found *m*/*z* 543.5101.

#### 3.2.8. 4-(Palmitoyloxy)octadecanoic acid (**18**)

Ozone was passed through a solution of compound **17** (521 mg, 1.00 mmol) in a mixture of CH_2_Cl_2_ (4.5 mL) and pyridine (0.5 mL) at −78 °C. Once the reaction solution had a persistent blue color, Ar was passed through until the solution was discolored, then dimethylsulfide (0.4 mL, 14.0 mmol) was added at 0 °C and the reaction mixture was left stirring for 1 to 2 h. Then, after concentration under reduced pressure, the residue was oxidized by a Pinnick oxidation. The desired product was obtained after purification by column chromatography (pet. ether 40–60 °C/EtOAc 8:2). White solid, 210 mg, m.p.: 129–131 °C, yield 39%; R_f_ value (dichloromethane:methanol = 9:1) 0.34; ^1^H-NMR (CDCl_3_) *δ* 4.91 (s, 1H, CH), 2.41–2.24 (m, 4H, 2 × CH_2_COO), 1.95–1.78 (m, 4H, 2 × CH_2_), 1.64–1.25 (m, 50H, 25 × CH_2_), 0.87 (t, *J* = 5.9 Hz, 6H, 2 × CH_3_); ^13^C-NMR (CDCl_3_) *δ* 178.5, 173.7, 72.9, 34.5, 34.1, 31.9, 30.0, 29.7, 29.6, 29.5, 29.4, 29.3, 29.2, 28.9, 25.2, 25.1, 22.7, 14.1; HRMS (ESI) exact mass calculated for [M − H]^−^ (C_34_H_65_O_4_^−^) requires *m*/*z* 537.4888, found *m*/*z* 537.4887.

#### 3.2.9. 2,2-d_2_-Pentadecanal (**5**)

To a flame-dried round-bottom flask under argon atmosphere, pentadecanal (226 mg, 1.00 mmol), D_2_O (1 mL) and triethylamine (0.01 mL, 0.10 mmol) were added and the mixture was left stirring in an oil bath at 100 °C for 1 h. Then, the mixture was allowed to warm to room temperature and after the addition of an aqueous solution of 1 N HCl, it was extracted with Et_2_O (2 × 10 mL). The organic layers were washed successively with an aqueous solution of 10% NaHCO_3_ (10 mL) and brine (10 mL) and then collected, dried over Na_2_SO_4_ and concentrated in vacuo. The aldehyde was immediately used for the next step without further purification. Colorless oil, 185 mg, yield 81%; R_f_ value (pet. ether:diethyl ether = 9:1) 0.37; ^1^H-NMR (CDCl_3_) *δ* 9.59 (s, 1H, CHO), 1.46–1.06 (m, 24H, 12 × CH_2_), 0.75 (t, *J* = 6.2 Hz, 3H, CH_3_); ^13^C-NMR (CDCl_3_) *δ* 201.9, 43.6–42.1 (m, CD_2_) 31.7, 29.5, 29.4, 29.3, 29.2, 28.9, 22.5, 21.7, 13.8.

## 4. Conclusions

In conclusion, we have developed a convenient enantioselective synthesis of biologically important 3-hydroxy fatty acids. Our methodology employs as the key-step the organocatalytic synthesis of chiral terminal epoxides using MacMillan’s third generation imidazolidinone organocatalyst thus ensuring high enantiomeric purity. The subsequent ring opening by vinylmagnesium bromide, followed by ozonolysis and oxidation leads to the target products. An approach for the synthesis of deuterated 3-hydroxy fatty acids is demonstrated, leading to deuterated derivatives, which can be useful in biological studies and in mass spectrometry studies as internal standards. Furthermore, a similar procedure using allylmagnesium bromide for the opening of epoxides leads to fatty γ-lactones and allows the synthesis of fatty acid asters of 4-hydroxy fatty acids.

## Supplementary Materials

Supplementary Materials are available online.
