# Supplementary material for: Enantioselective Organocatalysis-Based Synthesis of 3-Hydroxy Fatty Acids and Fatty γ-Lactones
Source: Molecules. 2019 May 31;24(11):2081. doi: 10.3390/molecules24112081 (PMC6600402; doi:10.3390/molecules24112081)
Supplement: Supplementary file 1 [file molecules-24-02081-s001.pdf]

Supplementary Data for

Enantioselective Organocatalysis-based Synthesis of 3-Hydroxy Fatty Acids and Fatty γ-Lactones

Asimina Bourboula, Dimitris Limnios, Maroula G. Kokotou, Olga G. Mountanea and George Kokotos*

Department of Chemistry, National and Kapodistrian University of Athens, Panepistimiopolis, Athens 15771, Greece

*Corresponding author. gkokotos@chem.uoa.gr (G. Kokotos)

Contents

Figure S1. Extracted-ion chromatogram of racemic 3-hydroxydodecanoic acid………………...2

Figure S2. Extracted-ion chromatogram of (*R*)-3-hydroxydodecanoic acid (**4a**)………………...2

Figure S3. Extracted-ion chromatogram of enantioenriched 3-hydroxypentadecanoic acid….. 3

Figure S4. Extracted-ion chromatogram of (*R*)-3-hydroxypentadecanoic acid (**4b**)…………..…3

Figure S5. Extracted-ion chromatogram of racemic 3-hydroxyhexadecanoic acid………………4

Figure S6. Extracted-ion chromatogram of (*R*)-3-hydroxyhexadecanoic acid (**4c**)………………4

Figure S7. Extracted-ion chromatogram of racemic 3-hydroxyoctadecanoic acid……………….5

Figure S8. Extracted-ion chromatogram of (*R*)-3-hydroxyoctadecanoic acid (**4d**)………………5

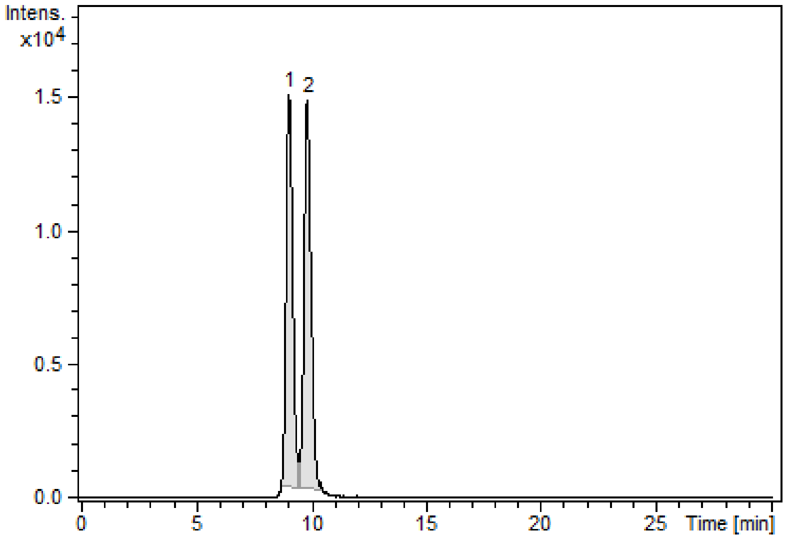

Figure S1. Extracted-ion chromatogram of racemic 3-hydroxydodecanoic acid.

#	RT [min]	Area	Chromatogram
1	9.2	298226	EIC 215.1634
2	9.9	319358	EIC 215.1634

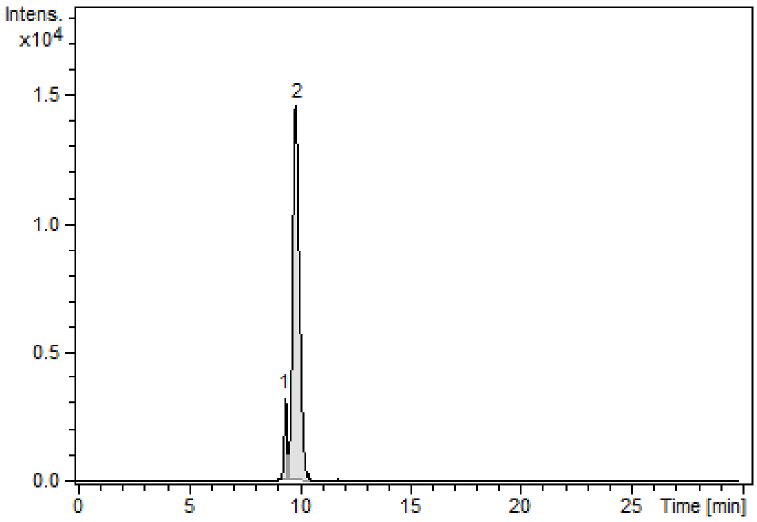

Figure S2. Extracted-ion chromatogram of (*R*)-3-hydroxydodecanoic acid (**4a**).

#	RT [min]	Area	Chromatogram
1	9.3	8851	EIC 215.1634
2	9.9	345182	EIC 215.1634

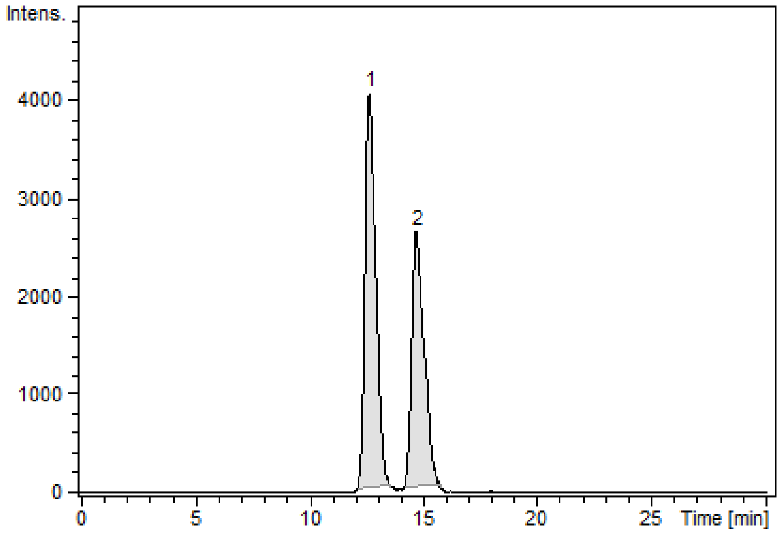

Figure S3. Extracted-ion chromatogram of enantioenriched 3-hydroxypentadecanoic acid (L-proline was used as the catalyst to prepare the starting epoxide).

#	RT [min]	Area	Chromatogram
1	12.7	130061	EIC 257.2109
2	14.7	98844	EIC 257.2109

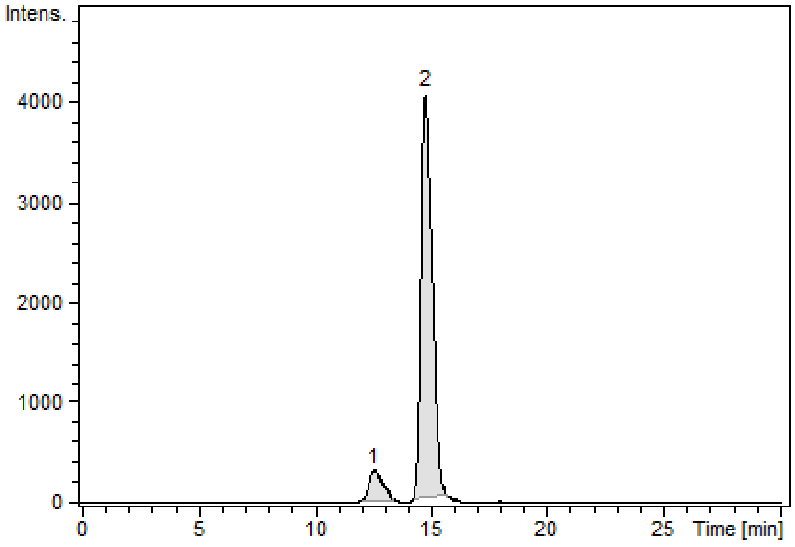

Figure S4. Extracted-ion chromatogram of (*R*)-3-hydroxypentadecanoic acid (**4b**).

#	RT [min]	Area	Chromatogram
1	12.7	6530	EIC 257.2109
2	14.7	138572	EIC 257.2109

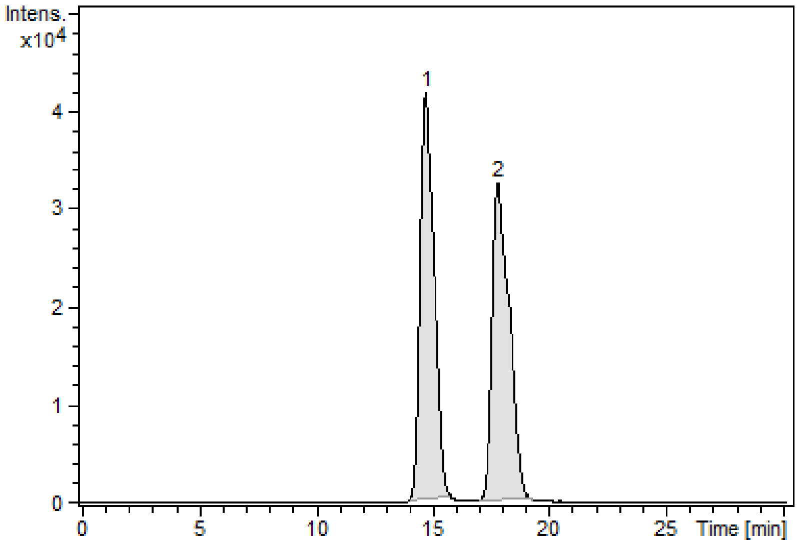

Figure S5. Extracted-ion chromatogram of racemic 3-hydroxyhexadecanoic acid.

#	RT [min]	Area	Chromatogram
1	14.7	1674014	EIC 271.2271
2	17.8	1623098	EIC 271.2271

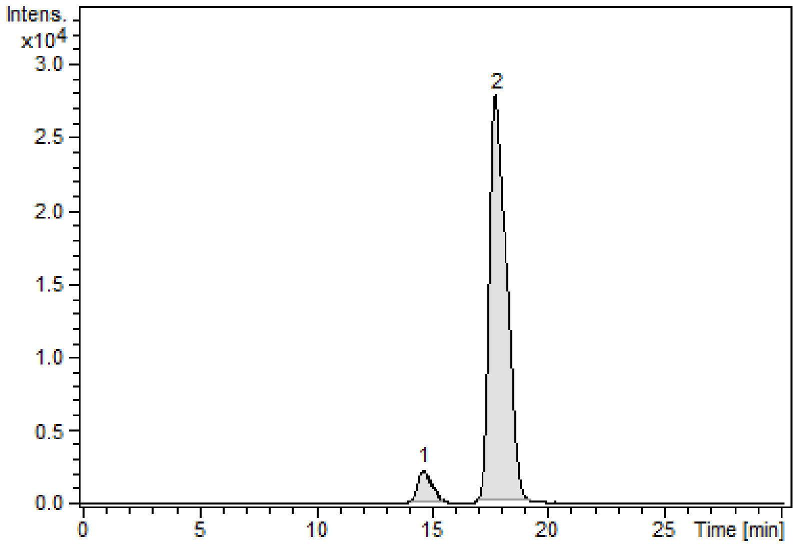

Figure S6. Extracted-ion chromatogram of (*R*)-3-hydroxyhexadecanoic acid (**4c**).

#	RT [min]	Area	Chromatogram
1	14.7	78949	EIC 271.2271
2	17.8	1377901	EIC 271.2271

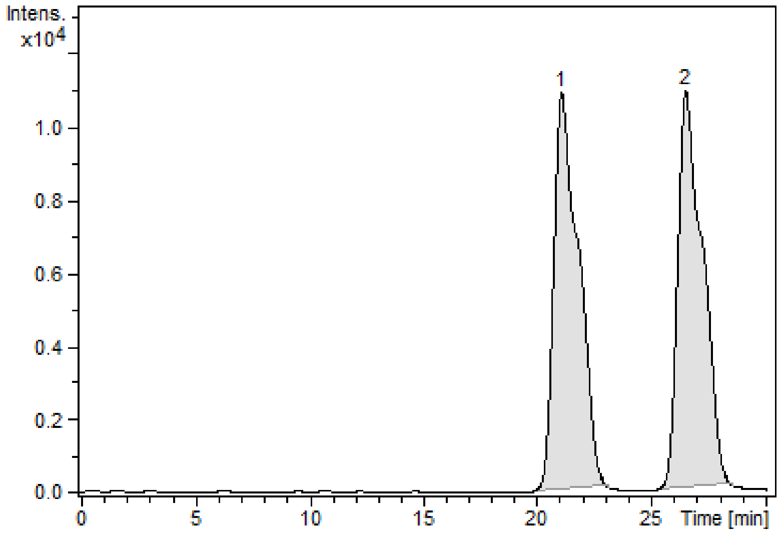

Figure S7. Extracted-ion chromatogram of racemic 3-hydroxyoctadecanoic acid.

#	RT [min]	Area	Chromatogram
1	21.0	801482	EIC 299.2613
2	26.6	798986	EIC 299.2613

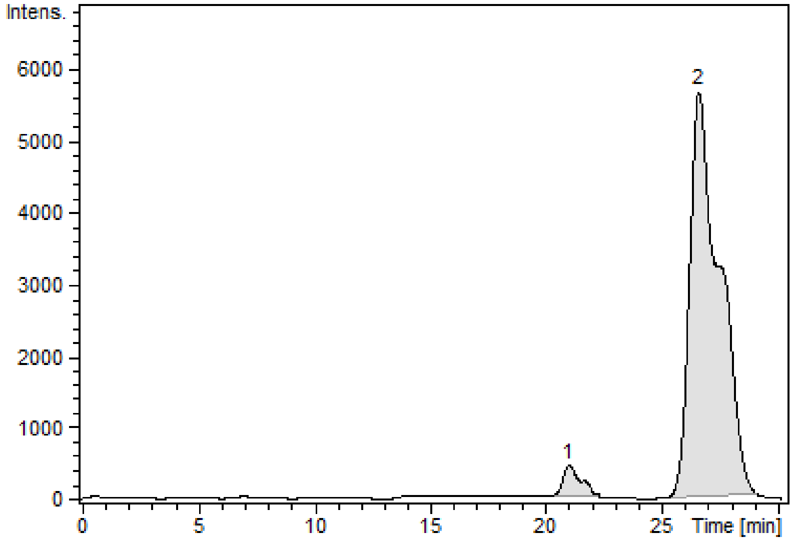

Figure S8. Extracted-ion chromatogram of (*R*)-3-hydroxyoctadecanoic acid (**4d**).

#	RT [min]	Area	Chromatogram
1	21.0	20585	EIC 299.2613
2	26.5	494047	EIC 299.2613
